# The Development of Nociceptive Network Activity in the Somatosensory Cortex of Freely Moving Rat Pups

**DOI:** 10.1093/cercor/bhw330

**Published:** 2016-12-26

**Authors:** P. Chang, L. Fabrizi, S. Olhede, M. Fitzgerald

**Affiliations:** 1 Department of Neuroscience, Physiology & Pharmacology, University College London, London WC1E6BT, UK; 2 Department of Statistical Science, University College London, London WC1E6BT, UK; 3 Current address: Department of Clinical and Experimental Epilepsy, UCL Institute of Neurology, London WC1N 3BG, UK

**Keywords:** development, EEG, neonatal, nociceptive networks, pain

## Abstract

Cortical perception of noxious stimulation is an essential component of pain experience but it is not known how cortical nociceptive activity emerges during brain development. Here we use continuous telemetric electrocorticogram (ECoG) recording from the primary somatosensory cortex (S1) of awake active rat pups to map functional nociceptive processing in the developing brain over the first 4 weeks of life. Cross-sectional and longitudinal recordings show that baseline S1 ECoG energy increases steadily with age, with a distinctive beta component replaced by a distinctive theta component in week 3. Event-related potentials were evoked by brief noxious hindpaw skin stimulation at all ages tested, confirming the presence of functional nociceptive spinothalamic inputs in S1. However, hindpaw incision, which increases pain sensitivity at all ages, did not increase S1 ECoG energy until week 3. A significant increase in gamma (20–50 Hz) energy occurred in the presence of skin incision at week 3 accompanied by a longer-lasting increase in theta (4–8 Hz) energy at week 4. Continuous ECoG recording demonstrates specific postnatal functional stages in the maturation of S1 cortical nociception. Somatosensory cortical coding of an ongoing pain “state” in awake rat pups becomes apparent between 2 and 4 weeks of age.

## Introduction

Pain is essential for survival and even newborn mammals need to process the sensory input from tissue damaging or threatening stimuli. However, while protective spinal and brainstem-mediated nociceptive reflexes are evident from before birth, the higher-level brain processing required for perception, learning, and memory of pain develops postnatally with increased exposure to the environment. In adult humans, it is now recognized that the conscious experience of pain arises from a dynamic change in a distributed network of brain activity (Davis et al. 2015; Kucyi and Davis 2015; Mano and Seymour 2015; Woo et al. 2015). The primary somatosensory cortex (S1) forms part of a pattern of cortical activity that is highly sensitive and specific to pain (Mancini et al. 2012), as manipulated by nociceptive input (Wager et al. 2013; Woo et al. 2015). Importantly, this activity is not simply a measure of noxious input, but tracks subjective pain more closely than the noxious stimulus itself. The somatosensory cortex appears to be related to a combination of nociceptive and affective aspects of pain, as distinct from evaluative and self-regulatory aspects (Wager et al. 2013; Woo et al. 2015). Since both newborn rat (Thairu 1971; Chang et al. 2016) and human S1 (Slater et al. 2006; Goksan et al. 2015; Verriotis et al. 2016) receives functional nociceptive thalamocortical inputs, this cortical region represents an appropriate brain area in which to begin to study the maturation of cortical pain networks.

Studies of the postnatal development of the somatosensory cortex have been largely confined to the rodent whisker barrel cortex, which have revealed considerable maturation of excitatory and inhibitory connections over the first weeks of life (Feldmeyer 2012; Dufour et al. 2016). At the end of the first postnatal week, intermittent burst activity in the whisker barrel cortex, driven by thalamic inputs, is replaced by an adult-like active cortical state and the emergence of organized sensory-evoked responses (Khazipov et al. 2004; Minlebaev et al. 2011) and a precise somatosensory map (Mitrukhina et al. 2015). These elegant studies, including the recently reported effects of alcohol and serotonin reuptake inhibitors (Lebedeva et al. 2015; Akhmetshina et al. 2016), are particularly important because they were performed *in vivo*, using head fixed rat pups following withdrawal of anesthesia.

Little is known about the formation of nociceptive networks in S1. The increasing complexity of S1 nociceptive evoked activity over the first four postnatal weeks in anesthetized pups suggests considerable network maturation in the first 3–4 weeks of life (Thairu 1971; Chang et al. 2016), a proposal that is supported by the changing energy and power of S1 oscillating signals within different frequency bands following noxious stimulation in pups under anesthesia (Devonshire et al. 2015; Chang et al. 2016). However, anesthesia alters cortical activity in an age-dependent manner (Granmo et al. 2013; Sitdikova et al. 2014; Chang et al. 2016) and a full understanding of the maturation of pain experience requires brain recording in awake animals. Real-time electroencephalography (EEG) and electrocorticogram (ECoG) recording from S1 in the awake, freely moving rat therefore offers an opportunity to investigate and understand the emergence of dynamic pain activity in the developing brain.

Successful EEG, ECoG, and local field potential recording in nonanesthetized, adult rats, and rat pups has been achieved in various behavioral studies (Lapray et al. 2008; Zayachkivsky et al. 2013; Sarro et al. 2014; Boulay et al. 2015; Xia et al. 2016). With respect to nociceptive processing, nociceptive laser evoked potentials have been recorded in the awake, adult rat S1 (Xia et al. 2016) and an increase in theta oscillations (4–8 Hz) has been observed in acute and chronic adult rat models, at rest (LeBlanc et al. 2014). However, long-term recording of S1 nociceptive and pain activity in awake, active pups without causing stress or impeding their healthy growth and maternal interactions represents a technical challenge. Here we report the results of high-quality long-term ECoG recordings from S1 using an open-source telemetry system (Chang et al. 2011), adapted for awake and active young rats, aged postnatal days 8–30 for up to 19 days. By continuously monitoring the changes in ECoG energy in the presence of a skin incision at different postnatal ages, we have mapped the maturation of nociceptive processing in S1. The results show that, following the arrival of thalamocortical input, cortical nociceptive networks in S1 go through distinct developmental stages, before displaying adult-like patterns of activity at 4 weeks of age.

## Materials and Methods

The objective of this study was to map the development of pain activity in the somatosensory cortex of awake, freely moving rat pups. To do this an EEG transmitter was implanted subcutaneously in rats of different postnatal ages from days (P) 6–30 and a recording electrode placed on the surface of the primary somatosensory cortex (S1) for a period of 1–18 days.

All experiments were performed in accordance with the United Kingdom Animal (Scientific Procedures) Act 1986. Reporting is based on the ARRIVE Guidelines for Reporting Animal Research developed by the National Centre for Replacement, Refinement and Reduction of Animals in Research, London, UK. Male Sprague-Dawley rats aged postnatal days (P) 6–30 were obtained from the Biological Services Unit, University College London. All animals were from the same colony, bred, and maintained in-house, and exposed to the same caging, diet, and handling throughout development. Rats were housed in cages of 5 age-matched animals (P30) or with the dam and littermates (P) 6–30 under controlled environmental conditions (24–25 °C; 50–60% humidity; 12 h light/dark cycle) with free access to food and water.

### Electrocorticography Recordings (ECoG) in Awake Animals

Rats were anaesthetized with 2.5–3% isoflurane (Abbot, AbbVie Ltd) in 100% oxygen (flow rate of 1–1.5 L/min) via gas anesthesia Mask (Model 906, David Kopf Instruments) from a recently calibrated vaporizer (Harvard Apparatus). Body temperature was maintained with heat blanket during surgery. An EEG transmitter (A3028B, Open Source Instruments, Brandeis Boston) (Chang et al. 2011) (see Supplementary Fig. S1) was implanted subcutaneously with the recording electrode positioned on the surface of the primary somatosensory hindpaw cortex contralateral to the stimulated side. Coordinates for P6–P14 were lateral 2.0 mm from midline and posterior 0.5 mm from the bregma; and for P18–P30 rats were lateral 2.5 mm from midline, and posterior 1 mm from the bregma (Khazipov et al. 2004; Paxinos and Watson 2013; Paxinos et al. 2013; Chang et al. 2016). A reference electrode was implanted in the skull over the cerebellum behind the lambda. The whole assembly was held in place with cyanoacrylate (Loctite). A subcutaneous injection of 0.1 mL bupivacaine around the wounds was given to animals for postsurgical pain management. The entire procedure was completed within 10–15 min. At the end of surgery, saline (1–2 mL) was administered subcutaneously. The animals were returned to their dam and littermates (<P21) or home cage (>P21) and closely monitored at least 3–4 h before returning to the animal house to allow recovery for at least 2–4 days after surgery. In two animals, the transmitter was chronically implanted at P11 until P29 for longitudinal data recordings.

All recordings were carried in a purposely designed recording room with temperature control (24–25 °C) in order to decrease ambient interference and improve the reception of the transmitting signals. ECoG signals were radio transmitted from the implanted transmitter to an antenna fixed a corner of the recording arena (A301501B) connected to a data receiver (A3018C) over distances up to 300 cm (filter bandwidth: 0.2 Hz to 160 Hz, 512 SPS with 16 bit resolution). The data were displayed and saved by Neuroarchiver Tool in LWDAQ Software (Open Source Instruments, Brandeis Boston). ECoG data were then imported into Matlab for further analysis. Only data during complete awake periods were further analyzed. Animals were carefully monitored daily and were euthanized at the end of experiment with pentobarbital (25 mg/kg).

### Experimental Protocol

**Figure 1.**
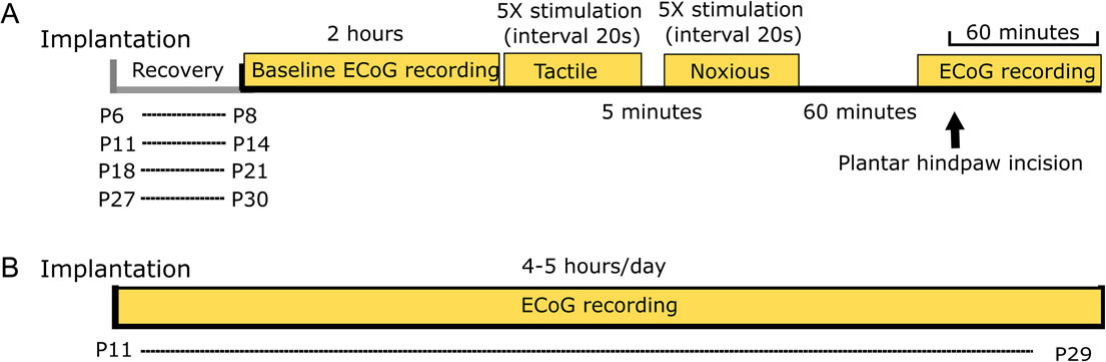
Experimental protocol. (*A*) Intracranial S1 ECoG recordings were performed in rats of different postnatal ages, 2–3 days after transmitter implantation. Baseline ECoG activity, evoked responses to cutaneous stimulation, and ECoG activity before and after plantar hindpaw incision were collected. A total of 36 rats were used. The full protocol was carried out on 25 rats. (*B*) In two animals ECoG was recorded continuously and collected for 4–5 h every day for 19 days.

This is illustrated in Figure 1. On the day of the recording, animals were transferred from the animal house to the recording room. After 1 h of acclimatization, ECoG was recorded 1) at baseline, 2) during cutaneous mechanical innocuous and noxious stimulation of the hindpaw, and 3) following a cutaneous injury (see Fig. 4). For the 2 animals with a chronic implant only baseline ECoG was recorded every day for 4–5 h.

#### Baseline ECoG

(P8, *n* = 5; P11, *n *= 10; P14, *n *= 9; P21, *n *= 10; P30, *n *= 9 and longitudinal P11–P29, *n* = 2) Below the age of weaning (<P21), rats were allowed to stay with their dam and littermates. At older ages (>P21), rats were allowed to freely navigate within their cage with their cage mates. Behavior was monitored during recording and periods of awake, active exploration noted.

#### Cutaneous Sensory Stimulation of the Hindpaw

(P8, *n *= 5; P14, *n *= 9; P21, *n *= 10; P30, *n *= 9) Cutaneous sensory stimulation was applied to one hindpaw. Dynamic tactile stimuli (brush) were delivered 5 times at 15–20 s intervals to the contralateral dorsal hairy skin of the hindpaw using a small camel hair paint brush (No. 2) applied in a proximo-distal direction from ankle to toes over 0.2 s. This was followed by interval break of 5 min. Next, noxious mechanical stimuli were delivered by pinching the mid portion of the dorsal hairy skin of hindpaw with serrated forceps for 0.2 s. Noxious pinch stimuli were applied 5 times at 15–20 s intervals.

#### Plantar Hindpaw Incision

(P8, *n *= 2; P14, *n *= 5; P21, *n *= 9; P30, *n *= 9) One hour after cutaneous sensory stimulation of the hindpaw, a subgroup of animals was anaesthetized and plantar hindpaw incision performed (Fig. 1). Under general anesthesia with 2% isoflurane in 100% oxygen (flow rate, 1–1.5 L/min), a midline longitudinal incision was made through the skin and fascia extending from the midpoint of the heel to the proximal border of the first footpad (to ensure the same relative length of incision at different ages) and the underlying plantar muscle elevated and incised as previously described (Brennan et al. 1996; Walker et al. 2009). Skin edges were closed with 5–0 nylon suture (Ethicon). The whole procedure took 3–5 min. After plantar hindpaw incision, rats were placed in a recovery chamber and allowed to recover from the general anesthesia before returning to their home cage. The animals rapidly recovered within 2 min after removal of the gas mask. The ECoG was continuously recorded before, during and up to 1 h after plantar hindpaw incision. Neuronal recording was time locked to the stimulation using a modified wireless transmitter with the two electrodes attached to the forceps. Pressing the forceps caused a signal, recorded simultaneously with the ECoG, in the same recording system.

### Data Analysis

#### Baseline ECoG

Analysis was conducted on recording periods when the animals were awake and active. In animals older than P13, this state is characterized by low-amplitude and high-frequency ECoG (Colonnese 2014). Epochs of 100 s were extracted from the continuous intracortical recordings. The power spectral density (PSD) of each 100-s ECoG epoch was estimated using the Welch's Overlapped Segment Averaging (WOSA) method as implemented in MATLAB. The time series is broke up in overlapping blocks, a periodogram is then calculated for each block using a window function and finally the individual periodograms are averaged together to provide an overall spectral estimate for the whole time series. In our analysis, we used $N_{B}=9$ blocks (of length $N_{S}$) with a 50% overlap and a Hamming window *h*. A sample mean PSD (${\overset{̅}{S}}_{x}\left(\omega \right)$, where ω denotes the frequency) at each postnatal age *x* was then calculated by averaging the PSDs (${\hat{S}}_{j}\left(\omega \right)$) of each animal *j* (with j=1,…,J) belonging to that age group or at each postnatal day for the longitudinal data. PSD data are displayed in decibel (dB).

#### ECoG Following Cutaneous Sensory Stimulation of the Hindpaw

Peak amplitudes (first positive to first negative peak) of tactile- and noxious-evoked potentials were computed for each animal (5 stimuli per animal). Data are given as grand mean ± standard error (number of stimuli × number of animals). Statistical analysis of peak amplitudes was performed using paired Student's *T*-test.

Continuous intracortical data were segmented into epochs of 10 s, from 5 s pre-stimulus to 5 s poststimulus. Epochs were then high-pass filtered at 0.5 Hz with a zero phase second order Butterworth filter. Time-frequency (TF) analysis was performed using a complex Morse wavelet transform (Olhede and Walden 2002). This allowed us to calculate a complex TF spectral estimate *W*(*a*,*b*) of the intracortical signal at each point (*a*,*b*) of the TF plane between 0.5 and 100 Hz (in logarithmic steps). We estimated the stimulus-induced energy changes time-locked to each stimulus at each postnatal age separately.

The TF changes in the ECoG energy induced by the stimuli were estimated as a group median. This was done by calculating the energy (i.e. modulus square) of the TF transform for each individual trial and then taking the median at each TF point (*a*, *b*) across all animals and trials. This value was normalized by the mean energy content of the baseline period at each frequency and represents the percentage energy changes time-locked to the stimulus. The statistical significance of these changes was tested against a threshold of 0.001. However, because these tests were conducted at any point (*a*, *b*) of the TF plane, false discovery rate (FDR) was used to correct for multiple comparisons (Benjamini and Hochberg 1995). The total number of independent tests was estimated by 1) calculating the number of independent tests at each frequency by dividing the length of the considered epoch by the length of the wavelet at that frequency (accounting for the correlation caused by the smoothing in time of the wavelet transform) and 2) adding those numbers across frequencies.

#### ECoG Following Plantar Hindpaw Incision

To assess the effect of skin incision on the ECoG frequency content, we first computed the sample mean PSD ${\overset{̅}{S}}_{2}\left(\omega \right)$ at 5, 30, and 60 min after plantar incision and recovery from anesthesia and the baseline ${\overset{̅}{S}}_{1}\left(\omega \right)$ at each postnatal age and then compared them. The PSD was again estimated using the WOSA method (Welch 1967) on 100 s long periods with $N_{B}=9$ blocks (of length $N_{S}$) with a 50% overlap and a Hamming window *h*. The sample mean PSD (${\overset{̅}{S}}_{x}\left(\omega \right)$) before (x=1) and after (x=2) plantar incision was then calculated by averaging the PSDs (${\hat{S}}_{j}\left(\omega \right)$) of each animal *j* (with j=1,…,J). PSD data are displayed in decibel (dB).

In order to conduct the comparison and quantify, its statistical significance at each frequency ω we need to define a frequency-specific test statistic under the null hypothesis that the spectral density after the plantar incision is the same as for the baseline observations. Under this null hypothesis

$$\frac{{\overset{̅}{S}}_{1}\left(\omega \right)}{S\left(\omega \right)}\sim a.\chi_{\nu }^{2},$$

and similarly for ${\overset{̅}{S}}_{2}\left(\omega \right)/S\left(\omega \right)$ (see Percival and Walden, 1993, p. 291), where *a* is a known constant. The degrees of freedom ν and the constant *a* of the specified distribution depend on: 1) the number of animals, 2) the number and length of the blocks, and 3) the windowing function to compute the PSD. As ν increases the $\chi^{2}$ distribution becomes increasingly Gaussian and we can approximate

$$\frac{{\overset{̅}{S}}_{1}\left(\omega \right)}{S\left(\omega \right)}\sim N\left(1,\frac{C\left(h\right)}{JN_{B}}\right),$$

and similarly for ${\overset{̅}{S}}_{2}\left(\omega \right)/S\left(\omega \right)$ In this expression, we calculate

$$C\left(h\right)=1+2\sum_{m=1}^{N_{B}-1}\left(1-\frac{m}{N_{B}}\right){\left|\sum_{t=1}^{N_{S}}h_{t}h_{t+\mathrm{mn}}\right|}^{2}.$$

Thus under the null distribution we compute the following normalized difference between ${\overset{̅}{S}}_{2}\left(\omega \right)$ and ${\overset{̅}{S}}_{1}\left(\omega \right)$:

$$T\left(\omega \right)=\sqrt{\frac{JN_{B}}{2C\left(h\right)}}\frac{{\overset{̅}{S}}_{2}\left(\omega \right)-{\overset{̅}{S}}_{1}\left(\omega \right)}{\overset{̅}{S}\left(\omega \right)}\approx^{d}N\left(0,1\right).$$

Direct calculations show that this quantity has approximately a Gaussian distribution standardized to mean zero and variance unity. In this expression, we have defined

$$\overset{̅}{S}\left(\omega \right)=\frac{1}{2}\left({\overset{̅}{S}}_{1}\left(\omega \right)+{\overset{̅}{S}}_{2}\left(\omega \right)\right),$$

The significance threshold for T(ω) was set at 0.05. However, because the test was repeated at each frequency, FDR was used to correct for multiple comparisons, accounting for the correlation between frequencies, and considering that Chi-square random variables can only exhibit positive correlation.

## Results

### Somatosensory ECoG Can Be Continuously Recorded in Awake Rat Pups

**Figure 2.**
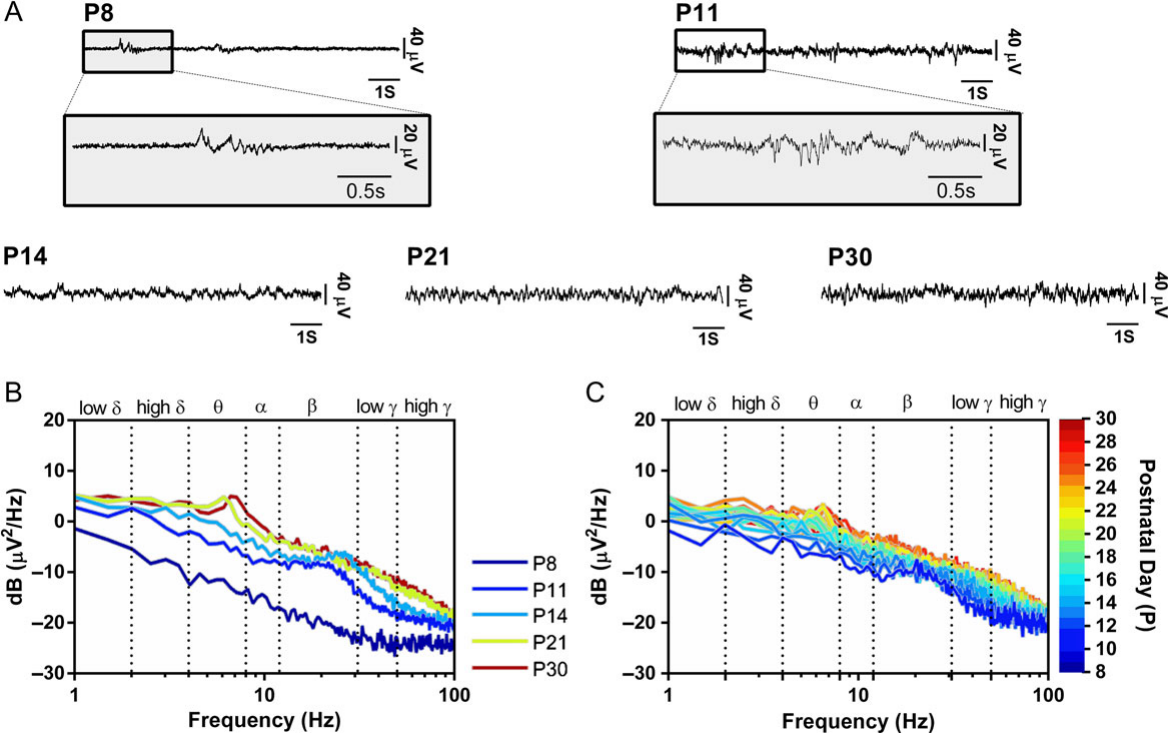
Background ECoG activity in the somatosensory cortex (S1) of awake, active rat pups changes with postnatal development. (*A*) Sample traces of ECoG signals recorded in S1 in awake animals at increasing postnatal ages from P8 to P30. (*B*) Energy PSD of S1 cortical activity at different postnatal ages (P8, *n* = 5; P11, *n *= 10; P14, *n *= 9; P21, *n *= 10; P30, *n *= 9). Overall energy increases with age. Distinct beta (20–30 Hz) activity in young rats, disappears in older rats and is replaced by distinct alpha/theta activity (4–8 Hz). (*C*) Longitudinal data showing the same developmental changes in PSD with age. ECoG activity was recorded every day from P11 through to P29 (*n *= 2). Further analysis is found in Supplementary Fig. S2.

Successful telemetric recordings from the somatosensory cortex hindlimb region were achieved in rat pups aged postnatal day, (P) 8, P11, P14, P21, and P30 (*n* = 5, 9, 10, 9) for up to 19 days. High-quality baseline S1 ECoG was recorded at all ages when animals were moving freely about the recording chamber (Fig. 2). Importantly, the implanted subcutaneous transmitter and cortical electrode had no adverse effects and pups developed normally within their litters, gaining weight at the same rate as their nonimplanted littermates (see Supplementary Fig. S1).

### Baseline ECoG Energy in the Awake Rat S1 Displays Distinct Age-Dependent Patterns

Baseline ECoG was recorded in the somatosensory cortex (S1) of awake, active rats, at each age. Sample traces in Figure 2*A* show bursting activity at P8 and P11, characteristic of the immature cortex (Colonnese et al. 2010; Minlebaev et al. 2011; Sitdikova et al. 2014), that is no longer present at P14, P21, and P30. Figure 2*B* shows PSD plots of baseline ECoG at each age. These plots show that the overall ECoG energy in active animals increases with age, between P8 and P21. This increase in energy with age is observed across all frequencies but is accompanied by specific changes in distinct frequency bands: a notable theta band (peaking at 6–7 Hz) at P21 and P30, which is not present at younger ages and a notable beta band (peak at 20–30 Hz) at P11 and P14, which is absent at older ages. Individual frequency plots show that ECoG energy at most frequencies increases until P14 and flattens out from P14 onwards, but that the theta and alpha energy continues to increase significantly between P14 and P30 (see Supplementary Fig. S2). These lower frequencies therefore contribute relatively more to the increase in total energy at later stages of development.

Figure 2 shows the same developmental pattern in the ECoG PSD in longitudinal recordings obtained from two pups, recorded continuously from P11 to P29. The marked beta energy, evident in very young pups, declines at P16–P18, at the same age as the marked theta energy emerges. Thus, just after 2 weeks of age, a distinct developmental change occurs in the relationship between energy and frequency of oscillations in S1.

### Differential Coding of Brief Tactile and Noxious Stimuli in the S1 of Awake, Active Rat Pups From P8

**Figure 3.**
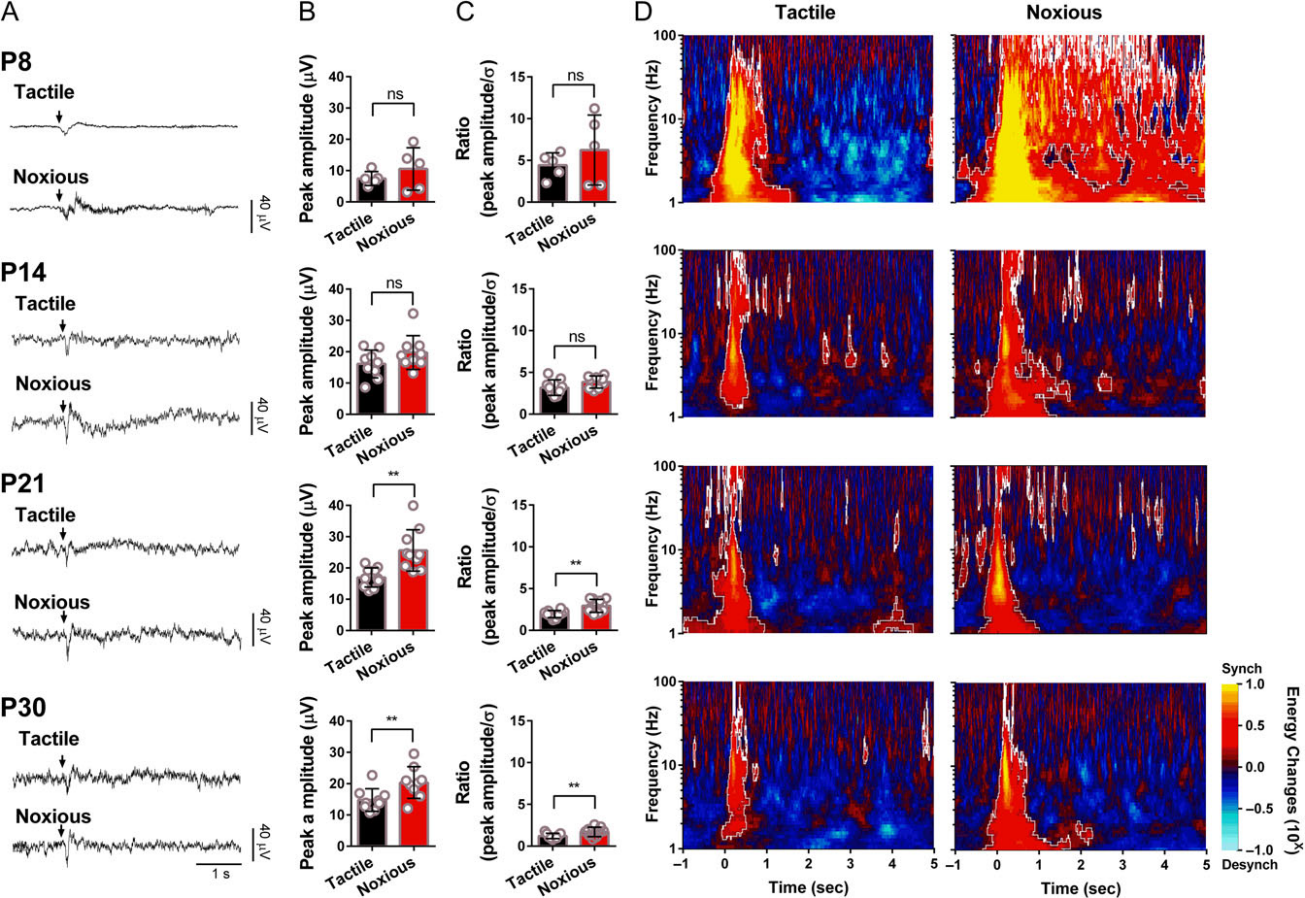
The development of ECoG activity recorded from S1 in response to contralateral hindpaw tactile and noxious mechanical cutaneous stimulation in awake rats. (*A*) Sensory-evoked ECoG responses following tactile and noxious stimulation in awake rats at at P8 (*n *= 5), P14 (*n *= 9), 21 (*n *= 10), and P30 (*n *= 9). Arrows indicate the stimulus onset. (*B*) Comparison of the peak amplitudes of sensory-evoked ECoG activity and (*C*) relative increases over baseline, following tactile and noxious stimulation in awake rats. Graphs show means ± SD. Statistical analysis was performed using Paired Student's *t*-test. * indicates *P* < 0.05, ** indicates *P* < 0.01, ns indicates nonsignificant. (*D*) TF decomposition of evoked ECoG activity following tactile and noxious stimulation in awake rats at P8, P14, P21, and P30. Results are displayed as increases and decreases of energy relative to baseline. Circumscribed areas indicate the significant changes in signal energy from baseline (*P* < 0.001).

To establish whether there is differential coding of innocuous and noxious cutaneous mechanical stimuli in S1, we recorded evoked potentials to dynamic brush and mechanical pinch stimulation of the hindpaw in awake active rats. Figure 3*A* shows typical event-related potentials (ERP; mean of 5 repeated stimuli) evoked by tactile and noxious stimulation at P8, P14, P21, and P30 (*n* = 5, 9, 10, and 9, respectively). Figure 3*B* shows the mean peak to peak amplitude of the potentials at each age. ERP to tactile and noxious stimulation do not differ at P8 and P14, whereas at P21 and P30, S1 noxious-evoked potential amplitudes are significantly greater than those of tactile-evoked potentials (mean SEP amplitudes: P21 tactile, 16.95 ± 3.05 μV; P21 noxious, 25.61 ± 6.60 μV, *t*_0.05(2),9_ = 3.574, *P* = 0.006; P30 tactile, 14.44 ± 3.59 µV; P30 noxious, 20.35 ± 5.03 µV, *t*_0.05(2),8 _= 3.749, *P* = 0.0056). Because of the lower baseline ECoG voltage fluctuations at younger ages, even though the absolute amplitude of the ERP increases with age, the proportional increase over baseline variance decreases (Fig. 3*C*).

To investigate energy changes in S1 ECoG, which are not reflected in voltage amplitude measures, we performed a TF decomposition of the signal. Figure 3*D* shows changes in mean energy above baseline across different frequencies (1–100 Hz) in the 5 s following hindpaw stimulation. Circumscribed areas represent significant energy changes compared with baseline.

At P8, tactile stimulation evokes a strong increase in energy at all frequencies in the first 500 ms poststimulus. Noxious pinch at this ages evokes a similar initial response but this is followed by a long-lasting increase in energy across all frequencies for the whole 5 s period. Note that the stimulus itself lasted a maximum of 200 ms. At P14 and older ages, the response to cutaneous stimulation is much reduced in energy, compared with P8, but pinch-evoked activity remains longer lasting than tactile-evoked activity at all ages, especially at delta frequencies (<4 Hz). The highest energy increases, particularly following pinch stimulation, were observed in the theta (4–8 Hz) range and at P14 and P21, pinch stimulation also evoked long latency increases in gamma energy.

### Specific Postnatal Developmental Stages in Nociceptive S1 Activity Following Skin Injury

**Figure 4.**
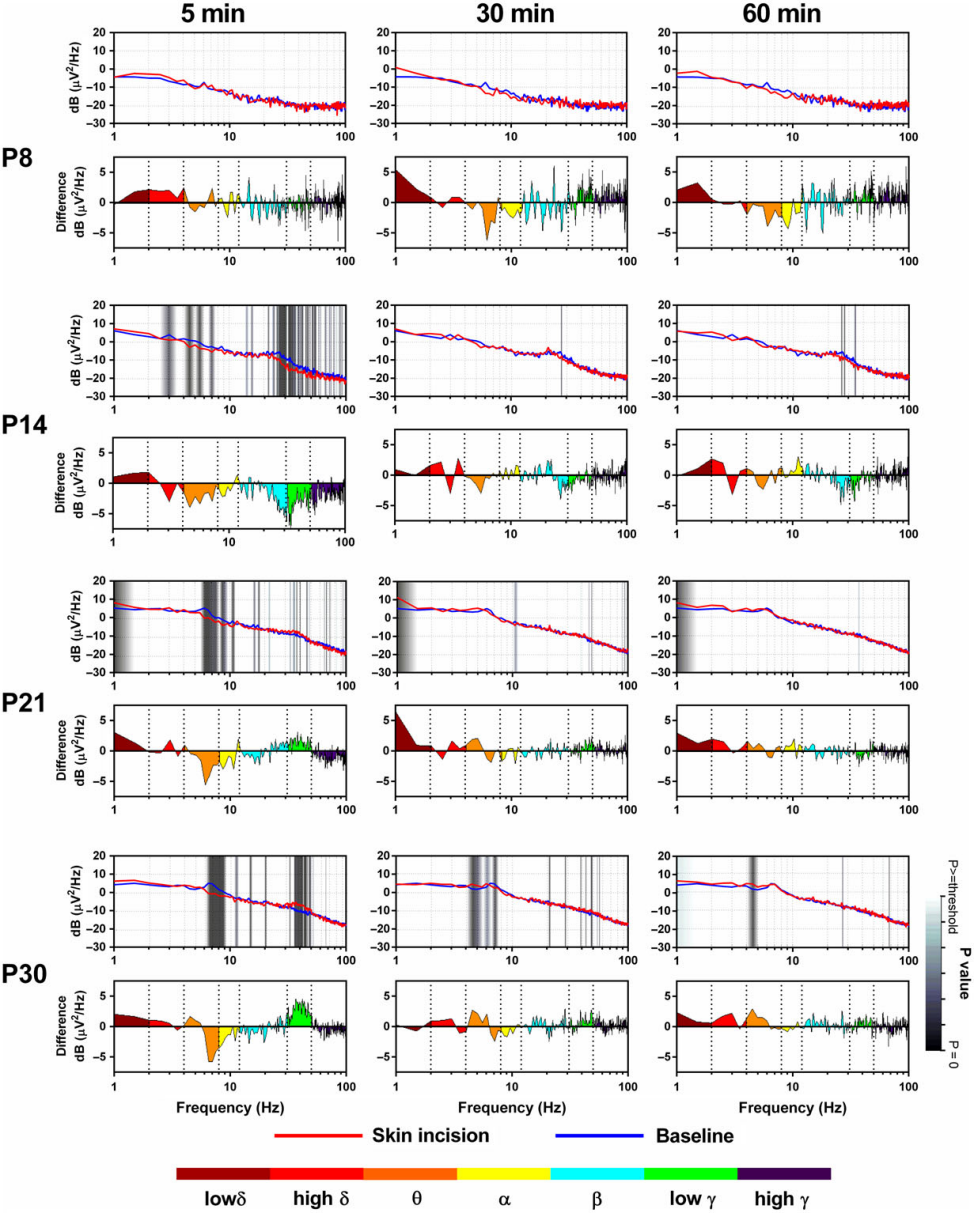
Changes in S1 cortical activity in S1 following plantar hindpaw skin incision in awake rats at postnatal days at 5, 30, and 60 min postincision. Two panels are presented at each age for each time point postincision. In the upper panels, the ECoG PSD at 5, 30, and 60 min after skin incision (red) is compared with background ECoG (blue) preincision. The lower panels show the spectral differences in different frequency bands (low *δ*: 1–2 Hz; high *δ*: 2–4 Hz; *θ*: 4–8 Hz; *α*: 8–12 Hz; *β*: 12–30 Hz; low *γ*: 30–50 Hz; high *γ*: 50–100 Hz, as color coded). Positive values indicate energy increases postincision, while negative values indicate energy decreases. Shaded vertical areas in the upper panels represent the statistical significance of these differences. P8 (*n* = 2), P14 (*n *= 5), 21 (*n *= 9), P30 (*n *= 9) rats.

We hypothesized that S1 cortical activity may better reflect the nociceptive experience of the animal rather than the stimulus intensity of a noxious stimulus. To test this, we recorded the ECoG from the S1 of awake, active animals that had undergone a hindpaw skin incision (Brennan et al. 1996). This injury is known to activate spinal nociceptive circuits within minutes (Shi et al. 2013) and induce hindpaw sensitivity for at least 24 h in both young and adult rats (Walker et al. 2009). We recorded continuous S1 ECoG for an hour following skin incision at each age. Figure 4 shows mean ECoG PSD plots in P8, P14, P21, and P30 awake, active rats at 5, 30, and 60 min after hindpaw skin incision compared with baseline. Significant differences between the 2 conditions are indicated by vertical-shaded areas on the plots.

#### Depression of S1 EcoG Energy by Skin Incision Injury at P8 and P14

Skin injury in the P8 neonate (*n* = 2) has no significant effect on the S1 EcoG energy postinjury (Fig. 4, top panel). At P14 (*n* = 5), energy is significantly reduced by the same injury experience. This reduction occurs 5 min postinjury and consists of significant decrease in delta (<4 Hz), theta (4–8 Hz), beta (20–30 Hz), and low gamma (30–50 Hz) energy (Fig. 4, second panel). By 30 and 60 min overall energy has effectively recovered but some depression in the beta and gamma band remains.

#### The Onset of a Distinct Skin Injury-Related Increase in S1 EcoG Gamma Energy at P21

At P21 there is a marked change in the S1 ECoG response to skin injury, compared with younger ages (Fig. 4, third panel). The early, significant energy decrease in the theta band, observed at P14, remains but is now accompanied by a significant increase in the gamma band that is not observed at younger ages. It is notable that the change in gamma energy has switched from a significant decrease at P14 to an increase at P21. The increase in gamma energy is short lived, however, and is no longer present at 30 and 60 min.

#### The Addition of a Distinct Skin Injury-Related Increase in S1 EcoG Theta Energy at P30

At P30 the increase in EcoG gamma energy following skin injury is stronger and longer lasting than at P21 and is significant at 5 and 30 min postinjury (Fig. 4, lower panel). The early decrease in theta energy, observed at P14 and P21, is still present at 5 min but is now switched to a significant increase in theta energy at 30 and 60 min postskin incision. P30 is the first age at which the effects of skin injury last for the full hour of recording.

## Discussion

Using real-time ECoG recording from the somatosensory cortex in the awake, freely moving rat pup, we have mapped the postnatal maturation of nociceptive activity in the developing brain. This is the first study of changes in SI cortical activity caused by a noxious skin injury over the first 4 weeks of life.

The increase in baseline ECoG energy in the somatosensory cortex (S1) with postnatal age reported here and the distinctive beta energy in very young rats is in agreement with previous reports of ECoG recording in awake P7–P11 pups (Zayachkivsky et al. 2013) and is comparable to that seen in premature human infants (Niemarkt et al. 2011). The relatively greater beta energy at P8 and P11 may arise from beta-oscillations, analogous to those reported in visual cortex at this age (Colonnese and Khazipov 2010), which are driven by thalamocortical inputs to synchronize activity in the superficial cortical layers and drive cortical circuit formation in early development. The relative increase in theta energy at P21 may signal the activation of longer range coupling between cortical pyramidal neurons (Bitzenhofer et al. 2015). These findings are strengthened by the fact that the same developmental pattern in ECoG power spectral density was obtained in cross-sectional and longitudinal recordings from P11 to P29.

To investigate the development of S1 nociceptive activity associated with a “pain state,” we used an established hindpaw skin incision model (Brennan et al. 1996). This defined skin injury rapidly excites primary afferents (Hämäläinen et al. 2002; Boada et al. 2012), activating spinal nociceptive neurons within minutes (Shi et al. 2013), releasing glutamate into the dorsal horn (Zahn et al. 2002) resulting in expansion of neuronal receptive field areas, spontaneous firing, and evoked spike activity in both young and adult rats (Vandermeulen and Brennan 2000; Ririe et al. 2008). Behavioral studies show that skin incision in adults and in neonatal rat pups induces pain behavior and hyperalgesia that lasts for days (Brennan et al. 1996; Walker et al. 2009) and that this injury when performed in the first week of life can have long-term consequences upon the development of spinal somatosensory circuits (Walker et al. 2016). The powerful effect of this injury upon spinal nociceptive activity in the youngest rats, contrasts with the lack of increase in somatosensory ECoG energy until the end of the third postnatal week.

At P8, hindpaw skin incision had no effect on S1 cortical activity, suggesting that nociceptive input has little effect upon SI networks at this age, despite clear evidence of strong spinal nociceptive activity (Fitzgerald 2005). At P14, the effect of the incision was to decrease energy across a range of frequencies including the gamma band. The predominant decrease in ECoG energy following skin injury at P14 could arise from decreased neuronal signaling or from desynchrony of cortical oscillations. Since spike timing is a crucial determinant of strengthening and weakening of synapses, desynchronization may enhance developmental plasticity (Feldman 2012). These data are reminiscent of the immediate cortical desynchronization observed in rat pups during maternal absence or by maternal behaviors, such as grooming and milk ejection (Sarro et al. 2014). While our recordings were always made in the presence of the dam, desynchronization may be a common mechanism underlying the shaping of cortical connections by noxious sensory stimulation or maternal deprivation (Nemeroff 2016). A normal background developmental transition from desynchronized to synchronized activity may also explain the increasing baseline ECoG energy with age observed here. In human infants synchrony between cortical activations also increases with age in the last 2 months before term (Koolen et al. 2016). However, skin injury also causes a transient but significant decrease in theta energy that is evident at all ages from P14 to P30 that cannot be explained by residual isoflurane from the surgery (MacIver and Bland 2014). This short lasting theta energy decrease develops earlier than the distinctive theta energy increases seen at later ages. It appears therefore that the decrease in theta energy is an important early component of somatosensory cortical activity, but may not be specific to nociception.

Skin injury at P21, on the other hand, caused a significant increase in gamma energy, 5 and 30 min postsurgery, and this was more pronounced at P30. This differs from the characteristic spontaneous gamma activity of the early neonatal cortex, which abruptly declines at P8 (Minlebaev et al. 2011; Khazipov et al. 2013), but is an evoked activity, not observed in the control period. Gamma activity has been highly associated with pain in adult rats (Li et al. 2010) and in humans it is strongly correlated with subjective pain intensity (Zhang et al. 2012). Furthermore, subjective perception of ongoing, tonic heat pain is selectively encoded by gamma oscillations in the human medial prefrontal cortex, differing fundamentally from that of objective stimulus intensity and of brief pain stimuli (Schulz et al. 2015). Skin injury at P30 also caused late onset increase in theta energy, 30 and 60 min postsurgery, which outlasted the gamma response. Gamma coherence has been associated with inter-regional communication within the brain, otherwise known as communication through coherence (Buzsáki and Draguhn 2004), but since the neural origins of the gamma oscillations recorded here is not known, the proposal that increases in gamma energy signals the beginning of long distance communications in pain networks should be viewed with caution (Buzsáki and Schomburg 2015). An increase in theta energy has been reported in awake adult rat somatosensory cortex following both capsaicin application to the skin and chronic nerve injury (LeBlanc et al. 2014, 2016b) and also after noxious thermal skin stimulation in anaesthetized adult rats (Devonshire et al. 2015). Theta and gamma energy, if comparable to human EEG data, could reflect the maturation of sensorimotor transformation of pain (Schulz et al. 2012) or increased attentional processing and enhanced saliency of pain-related signals (Hauck et al. 2007) as well as subjective pain intensity (Zhang et al. 2012; Dufort Rouleau et al. 2015).

These results should be viewed in the context of the overall postnatal development of the rat cortex. The second week of life represents a pivotal point in the transition from immature to mature cortex in terms of cell growth, synaptogenesis, mechanisms of cortical plasticity, NMDA activity and GABA hyperpolarization (Dehorter et al. 2012), marking a time when cortical activity becomes strongly modulated by behavioral states (Cirelli and Tononi 2015). The emergence of a “pain state” in the somatosensory cortex, characterized by initial suppression in slow and enhancement of fast activities, which emerges and matures through the third and fourth postnatal weeks, has similarities with the transition from quiet awake to active explorative states, which also emerge at this age. The results reported here are based upon on ECoG recordings obtained while the pups were awake and actively exploring, both pre- and poststimulus, in order to minimize the general effects of arousal, but the 2 may share some of the same mechanisms.

The data support the conclusion that somatosensory cortical activity associated with injury-induced pain experience requires the maturation of cortical networks beyond that required for the ERP that follow brief tactile or noxious pinch of the hindpaw skin, which are also observed in anesthetized rat pups (Chang et al. 2016). Modality-specific responses to tactile and noxious hindpaw stimulation can be recorded from nociceptive specific and wide dynamic range neurons in the neonatal rat dorsal horn in the first week of life (Fitzgerald 2005) and while ascending nociceptive projections to thalamus develop early, functional nociceptive inputs to brainstem nuclei involved in descending control are not present until the second postnatal week (Schwaller et al. 2016). The delayed, activity-dependent maturation of dorsal horn inhibitory signaling results in large receptive fields and prolonged firing to noxious stimulation in the neonatal dorsal horn (Koch and Fitzgerald 2013), which likely contribute the long-duration noxious ERPs recorded here at P8 and P14. It is likely that pain-related cortical network oscillations have a different origin from nociceptive ERPs (Li et al. 2010). The reason for the functional delay in pain-related oscillations is not known but may relate to the development of intracortical connections and with other key brain regions involved in pain processing. This is consistent with the delayed onset of interhemispheric and higher-order cortical somatosensory stimulus BOLD responses following electrical forepaw stimulation at P13 (Colonnese et al. 2008). Many areas involved in pain processing in adults are maturing over this time: such as the insular cortex (Gogolla et al. 2014) and the amygdala (Tallot et al. 2016). Important too, may be the delayed maturation of the corticofugal control of brainstem nuclei, responsible for controlling sensory inputs and motor behavior (Schwaller et al. 2016).

A limitation of this study is the emphasis on the primary somatosensory cortex (S1), which forms only a part of the distributed dynamic network that, in adult man, underlies the conscious experience of pain (Davis et al. 2015; Kucyi and Davis 2015; Mano and Seymour 2015; Woo et al. 2015). However, the primary somatosensory cortex is key part of that network and is highly sensitive and specific to pain across individuals, both human (Mancini et al. 2012; Wager et al. 2013; Woo et al. 2015) and rodent (Hubbard et al. 2015; Amirmohseni et al. 2016) and as such, represent a rational cortical location for such a developmental analysis. Differences in the S1 ECoG power spectral density across postnatal ages could have been affected by changes in the reference position as the animal grows and interference from movement but these problems were minimized with the current recording setup.

Future studies could include multiple recording sites across other brain regions but it is important that the rat pups develop normally and are awake, moving around and free of the stress during recording. Head fixing or tethered recordings, which are commonly used in awake cortical recordings, can be stressful and blunt pain perception (Low et al. 2016). The stimulus was also confined to noxious mechanical stimulation in the hindpaw and it would be important to explore other nociceptive modalities and body regions in the future. The effects of stimulus evoked hyperalgesia and analgesic efficacy, as defined by behavioral tests, upon cortical activity could also be investigated (LeBlanc et al. 2016a), as could any sex differences in immature nociceptive brain circuits.

The results presented here may be translatable to cortical pain processing in human infants (Fitzgerald 2015), but rats are an altricial species and comparisons in cortical development between rodents and humans are dependent upon many factors specific to particular central nervous system regions and circuits (McCutcheon and Marinelli 2009). Nevertheless, homologous brain rhythms have been recorded in the adult rat, primate, and human somatosensory cortex (Fransen et al. 2016). Using comparative anatomical data, the P14 rat cortex has been proposed to be equivalent to that of a 29-week gestation human infant (Clancy et al. 2001), while a more recent algorithm, based on the maturation of visual cortical synaptic proteins, suggests that a P11 rat pup is equivalent to a term human infant (Pinto et al. 2015). Scalp EEG recording in preterm human infants has demonstrated that clinically required noxious skin stimulation evokes specific nociceptive ERPs from 30 to 35 gestational weeks onwards gradually replacing the immature nonspecific burst responses (Fabrizi et al. 2011). Time frequency analysis of cortical activity in full-term infants shows that nociceptive stimuli are encoded differently from that in adults (Fabrizi et al. 2016) but more research is required to understand the maturation of pain representation in the human infant brain. Oscillations and their synchronization are important correlates of neuronal processing, and coordination of neural activity into whole-brain functional networks can provide valuable measures for the assessment of brain functions. Changes in ECoG power spectral density during postnatal development reflect the development of functional circuitry in the brain. Real-time ECoG recording in the awake, freely moving rat offers an opportunity to understand the emergence of nociceptive processing and the representation of “pain states” in the developing brain.

## Supplementary Material

Supplementary material can be found at: http://www.cercor.oxfordjournals.org/.

Supplementary Data
