# Supplementary material for: The Development of Nociceptive Network Activity in the Somatosensory Cortex of Freely Moving Rat Pups
Source: Cereb Cortex. 2016 Dec 26;26(12):4513–23. doi: 10.1093/cercor/bhw330 (PMC5193146; doi:10.1093/cercor/bhw330)
Supplement: Supplementary Data [file supp_26_12_4513__index.html]

The Development of Nociceptive Network Activity in the Somatosensory Cortex of Freely Moving Rat Pups — The Development of Nociceptive Network Activity in the Somatosensory Cortex of Freely Moving Rat Pups — Supplementary Data 

# The Development of Nociceptive Network Activity in the Somatosensory Cortex of Freely Moving Rat Pups

## Supplementary Data

Supplementary Data

- Supplementary Data - docx file
