# Supplementary material for: The Development of Nociceptive Network Activity in the Somatosensory Cortex of Freely Moving Rat Pups
Source: Cereb Cortex. 2016 Dec 26;26(12):4513–23. doi: 10.1093/cercor/bhw330 (PMC5193146; doi:10.1093/cercor/bhw330)
Supplement: Supplementary Data [file supp_bhw330_Supplementary_Materialssecondsubmission3816.docx]

Supplementary Materials

**Fig. S1. The impact of transmitter implantation on the postnatal growth in rats.**

The transmitter was encapsulated in epoxy and silicone was subcutaneously implanted in SD rats at postnatal age 11 and the pups were monitored for the next 19 days. The transmitter was placed beneath an incision on the back, the leads were run under the skin to the scalp, where M0.5 screws (diameter: 0.5 mm and length 0.6 mm) act as electrodes through skull and record the surface EEG on the surface of the somatosensory cortex. **A**. A photograph of the transmitter. **B**. Summary of the transmitter properties. **C-E**. There was no significant impact on postnatal growth, measured as **C.** body weight **D**. body width and **E**. body length from postnatal day (P) 11 to 29 days after implantation. Data are shown as means ± SD. (N=2 for control; N=7 for implantation). **F**. Photographs of rats at postnatal day (P) 14, 21, and 28, after implantation at P11.





**Fig. S2. The changing energy of baseline S1 cortical activity with postnatal development.**

Plots show total energy and the energies of each frequency component of SI cortical activity. The energy values were obtained using Welch power spectrum analysis of 100s EEG segments recorded at different ages. Frequency bands are defined as: low δ: 1-2 Hz; high δ: 2-4 Hz; θ: 4-8 Hz; α: 8-12 Hz; β: 12-30 Hz; low γ: 30-50 Hz; high γ: 50-100 Hz. Graphs show means ± SD. Statistical analysis was performed using one-way ANOVA with post hoc Tukey's multiple comparison test. * indicates P<0.05, ** indicates P<0.01, and *** indicates P<0.001. (P8, n=5; P11, n=10; P14, n=9; P21, n=10; P30, n=9).
